# Multilevel Impacts of Iron in the Brain: The Cross Talk between Neurophysiological Mechanisms, Cognition, and Social Behavior

**DOI:** 10.3390/ph12030126

**Published:** 2019-08-29

**Authors:** Ana Ferreira, Pedro Neves, Raffaella Gozzelino

**Affiliations:** 1 Centro Interdisciplinar de Ciências Sociais (CICS.NOVA), Faculdade de Ciências Sociais e Humanas da Universidade NOVA de Lisboa (NOVA FCSH), 1069-061 Lisbon, Portugal; 2 School of Business and Economics, NOVA University of Lisbon, 2775-405 Lisbon, Portugal; 3 Chronic Diseases Research Center (CEDOC)/NOVA Medical School, Universidade NOVA de Lisboa, 1180-052, 1150-082 Lisbon, Portugal

**Keywords:** iron, brain, neurophysiology, cognition, social behavior

## Abstract

Iron is a critical element for most organisms, which plays a fundamental role in the great majority of physiological processes. So much so, that disruption of iron homeostasis has severe multi-organ impacts with the brain being particularly sensitive to such modifications. More specifically, disruption of iron homeostasis in the brain can affect neurophysiological mechanisms, cognition, and social behavior, which eventually contributes to the development of a diverse set of neuro-pathologies. This article starts by exploring the mechanisms of iron action in the brain and follows with a discussion on cognitive and behavioral implications of iron deficiency and overload and how these are framed by the social context. Subsequently, we scrutinize the implications of the disruption of iron homeostasis for the onset and progression of psychosocial disorders. Lastly, we discuss the links between biological, psychological, and social dimensions and outline potential avenues of research. The study of these interactions could ultimately contribute to a broader understanding of how individuals think and act under physiological and pathophysiological conditions.

## 1. Introduction

Iron is a vital element for most organisms. Iron actively participates in many physiological processes, such as oxygen transportation, cellular respiration, energy production, cell growth and differentiation, DNA synthesis, and more. Its ability to transfer electrons among different substrates is a property that renders this metal essential for life. However, it also implies the existence of fine-tuned regulatory mechanisms strictly controlling iron homeostasis, since minimum disruption of iron balance significantly alters organs’ functionality [1]. Many mechanisms contribute to maintain iron homeostasis, with a vast number of genes being involved in iron uptake, storage, and recycling. So far, there is no description of physiological mechanisms of iron excretion.

In the specific case of the brain, which is a metabolically active organ and particularly sensitive to changes in iron homeostasis [2], there are still many uncertainties. This is the case since the distribution and roles of genes devoted to iron homeostasis are still under discussion. Current knowledge points at iron crossing the blood brain barrier (BBB) as iron-loaded transferrin (holotransferrin, HTf) complexes. In addition, binding of iron to transferrin receptors on capillary endothelium results in their subsequent internalization by forming endocytic vesicles. Iron is then pumped out via the expression of divalent metal transporter 1 (DMT1) in its ferrous form. In the cytosol, ceruloplasmin is the oxidase responsible for converting this metal into its ferric form, which can be exported in the extracellular space by ferroportin [3]. In the brain, disruption of iron homeostasis significantly impairs oxidative metabolism of neural cells, with dramatic consequences for synaptic plasticity, myelination, and synthesis of neurotransmitters [4,5]. This means that both iron deficiency and overload are associated with disruption of neurophysiological mechanisms, which were previously associated with impaired cognition and altered social behavior, as described in the following sections of this paper.

**Figure 1:**
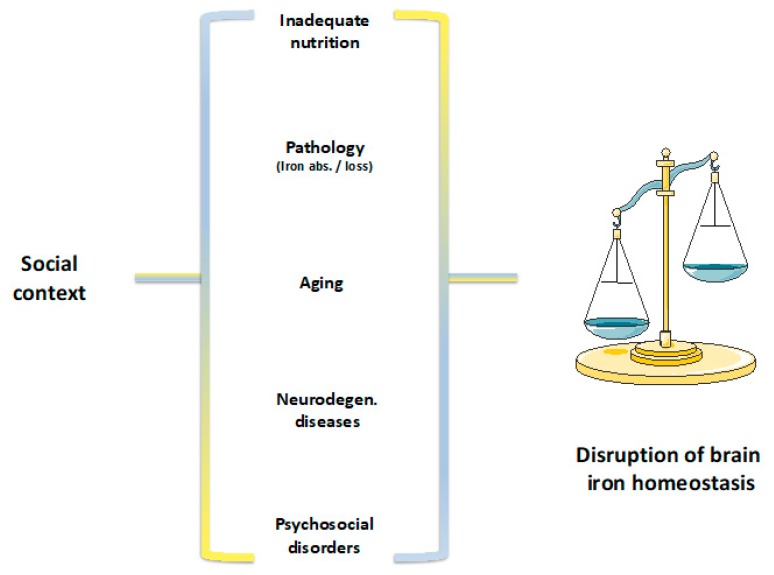
Processes underlying disruption of brain iron homeostasis. Social context, integrating structural (e.g., socio-economic context, life trajectories, socio-economic status, education, knowledge and skills, norms and values), social interactions (e.g., the specific context of interactions; who is present) and individual dimensions (e.g., appraisal, perceptions, motivations and expectations, and reflexivity) frames the pathophysiological mechanisms of an inadequate nutrition (e.g., poor diets, excessive milk intake, vegetarian diets), pathologies associated with inadequate iron absorption (e.g., Celiac disease) or excessive iron loss (e.g., blood loss, parasitic infections), aging, neurodegenerative diseases (e.g., Alzheimer’s and Parkinson’s disease) or psychosocial disorders (e.g., chronic social defeat stress, anxiety, depression). All these conditions are associated with a disruption of brain iron homeostasis.

Regarding iron deficiency, which is the most common nutrient deficiency worldwide [6,7], the absence of mobilizable iron stores and compromised supply of iron to tissues, including erythrocytes, can identify it. The most severe form of iron deficiency, i.e., iron deficiency anemia, is characterized by the levels of iron associated with hemoglobin lower than two standard deviations of the distribution of mean hemoglobin in a population of the same age, sex, living at the same altitude, and without any other pathologies [6]. The causes underlying iron deficiency are diverse and include: inadequate oral iron intake, resulting from poor diets, excessive milk intake or vegetarian diets, inadequate iron absorption, as a result of Celiac disease and others, or excessive iron loss, mainly occurring via blood loss or as a result of parasitic infection (Figure 1) [6,8]. Although individuals can suffer from iron deficiency at any stage in life, the most severe consequences of iron deficiency are present in early childhood, which is a period of rapid growth and high needs for iron intake. These range from the disruption of neurophysiological mechanisms, to impaired cognitive and altered social behavior. Most importantly, iron deficiency has been reported in low-income and high-income countries, with increased prevalence of iron deficiency in lower socio-economic groups. These have been concomitantly characterized by worse eating habits, lower levels of education, and acknowledgement of the impacts of nutritional patterns in physical and cognitive development and health [9,10,11,12,13,14,15]. In this population, dietary iron intake was shown not only to be influenced by personal taste, attitude, and knowledge, but also by food access and cost, which render low–income groups at a multiple disadvantageous position from the go ahead [9,16]. Altogether, this could explain the higher risk for iron deficiency in lower socio-economic groups.

Currently, the World Health Organization (WHO) recommends iron replacement therapies as an initial intervention to address iron deficiency [17]. These supplementations have been successful in the control of anemia and iron deficiency in different age groups. However, the long-term deficits in motor and cognitive functions, as well as the modifications in social behavior, were mostly shown to persist and deserve great attention and debate [8,18,19,20]. Several reasons can account for the absence of (or very small, in a few cases) motor, cognitive, and behavioral effects. On the one hand, methodological insufficiencies (i.e., study design limitations, indirectness of presented evidence, imprecisions, or reporting bias) and heterogeneity of intervention protocols (precluding a straight comparison among studies) can partly account for the reported inconsistences. On the other hand, these studies mostly assume that correction of the nutrient deficiency per se, would suffice to address long-term motor and cognitive impairments, as well as modifications in social behavior. However, as we will discuss in the following sections of this paper, one can argue that there seems to be an overlap between the biological effects of a nutrient insufficient diet and its effects on neurophysiological mechanisms and cognition, as well as a potential absence of social stimuli to promote adequate physical and cognitive development. Most studies do not take into account, or analyze superficially at most, the interactions between biological, psychological, and social dimensions of a nutrient deficiency, subsequent multi-organ disruption of iron homeostasis, and its impacts on motor deficits, cognitive impairments, and modifications of social behavior. Future research should address these interactions much more profoundly, by integrating the immense knowledge patrimony of diverse scientific disciplines, and characterizing the role of each dimension, their interactions, and potential contributions for the multiple reported outcomes. This has not been previously done.

On the opposite pole of iron deficiency, we have iron overload, which is present, for instance, in the aging population and in protein misfolding neurodegenerative diseases, such as Alzheimer’s or Parkinson’s disease (AD and PD, respectively) (Figure 1) [21]. In the specific case of AD and PD patients, mental decline, as evaluated by the Dementia Rating Scale and the total Frontal Systems Behavior Scale, was shown to be present [22]. More specifically, AD patients have difficulties in storing information and learning new data. PD patients are unable to prevent memory retrieval and mind forgetfulness, which are characteristics that can alter the cognitive and behavioral profiles of these subjects [23]. Lastly, AD and PD progression are associated with depression, anxiety, psychotic symptoms, and sleep disturbances [24,25] and likely impacted by a dysregulation of neural circuitries. This dysregulation can be triggered by the brain iron accumulation present in these patients.

In psychosocial disorders like chronic social defeat stress, anxiety, and depression, iron’s ability to modulate the levels of neurotransmitters released in the synaptic clefts was shown to contribute to commonly observed cognitive impairments and behavioral modifications [26]. These conditions are also characterized by changes in both structure and function of the prefrontal cortex, where the expression of genes regulating iron metabolism appears to be significantly altered. This means that brain iron accumulation is likely involved in the outcome of stress-induced depression, and pathophysiological changes typical of mood and anxiety disorders (Figure 1) [27]. In the body, 80% of iron is incorporated within the protoporphyrin ring of heme molecules, which is the prosthetic group of heme proteins. Among heme proteins, hemoglobin is the most abundant [2,28]. Interestingly, studies in animal models revealed an increase in hemoglobin gene expression in the brain of animals that were exposed to certain levels of stress. Even though the function of hemoglobin in the brain is still unknown, it has been proposed to control the vascular tone. If that is the case, its increase might protect neurons against stressful social encounters that could, otherwise, compromise brain functions by damaging its vascular system [29]. In mice, the expression of genes that directly regulate iron metabolism was also found to be changed under stressful conditions. A reduction in the iron importer Transferrin Receptor 1 was observed in response to stress. This reduction could possibly prevent further intracellular iron entry, which would subsequently harm neurons in the pre-frontal cortex, i.e., an area that is mainly involved in conscious decision-making and social action [27]. Conversely, an increased expression of lipocalin 2, which is an iron transporting protein that regulates the morphology and excitability of neurons in the hippocampus and amygdala when mice are exposed to stress [27,30], was associated with a number of modifications in behavioral responses, depression, neuronal excitability, and anxiety [31]. The reasoning for this is that, in the absence of a neutralizing system, an increased brain iron accumulation is capable of promoting oxidative stress and neuronal death, processes that could be undermining cognition, which potentially frames social behavior and eventually leads to pathological development [32].

Given the increasing life expectancy of the world’s population, as well as the increasing prevalence of both mentioned neurodegenerative and psychosocial disorders, the study of iron overload and of its etiology is of the utmost relevance. Since the role of iron in neurodegenerative pathologies was the focus of several reviews [3,33,34,35,36], for the purpose of this article, we will only address its impacts in psychosocial disorders. As for iron deficiency, we will review previous studies, and discuss the pressing need for research integrating the causes underlying iron overload and its impacts on neurophysiological mechanisms, as well as on concomitant modifications of motor, psychological, and social processes of populations affected by excessive iron.

**Figure 2:**
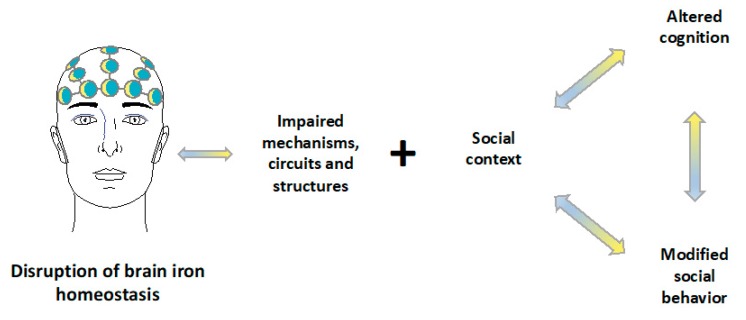
Impacts of disruption of brain iron homeostasis. Dysregulation of brain iron metabolism profoundly affects neurophysiological mechanisms (e.g., neurotransmitter synthesis and metabolism, axon myelination, neural transmission), neural circuits (e.g., mesocortical, mesolimbic, nigrostriatal, and tuberohypophyseal dopaminergic pathways) and brain structures. In the presence of a social context that does not provide stimuli to promote regular cognition and normative social behavior, disruption of brain iron homeostasis is associated with altered cognition (e.g., executive function, attention, memory) and modified social behavior (e.g., functional isolation, wariness, and hesitance behaviors).

This paper argues that brain iron dysregulation has profound neurophysiological impacts and is associated with cognitive and behavioral modifications that could be further exacerbated by social contexts that do not promote regular cognitive function and normative social behavior (Figure 2). Hence, this review starts by exploring the mechanisms of iron action in the brain and presents an overview of iron metabolism and of its cytotoxic mechanisms of action. A discussion of cognitive, behavioral, and pathological implications of iron deficiency and overload, and how these can be framed by the social context, will follow. The links between biological, psychological, and social dimensions will be explored and potential avenues of research will be pointed out.

## 2. Iron in the Brain

It has been previously shown that the brain is particularly sensitive to changes in iron homeostasis [2,4,5]. This is the case since iron is critical for maintenance of the high metabolic and energetic requirements of neuronal tissues, neurogenesis, axon myelination, synaptic development, neurotransmitter synthesis, and metabolism [37]. As such, the brain requires considerable amounts of iron, particularly in periods of rapid growth, such as early childhood. These levels are mainly up-taken from the blood, which is the source of brain iron through adult life, when its up-take is considerably lower. In addition, presenting a considerable variation is the distribution of iron in the brain. More specifically, the highest concentrations of iron are found in the basal ganglia, particularly in the substantia nigra, globus pallidus, nucleus caudate, red nucleus, and putamen [38]. Since brain iron is mainly up-taken from the blood, the brain iron status is directly affected by peripheral iron levels and nutrition. In fact, iron deficiency, which is the most prevalent nutrient deficiency worldwide, has been proposed to impact motor function, cognition, and social behavior [26]. In contrast, accumulation of iron in the brain has been associated with aging, neurodegenerative diseases, and psychosocial disorders [26,32,39]. This raises the question about potential adverse effects induced by iron food biofortification, which, in the long term, might contribute to tissue iron accumulation and, ultimately, mediated damage. Nevertheless, iron overload was mostly studied in animal models and the results might not be fully translatable into human subjects, especially in studies exposing mice to non-physiological levels of dietary iron. It is not surprising that behavioral dysfunctions and neurological effects appear when animals are fed with exacerbated concentrations of this nutrient [26,40]. Similar manifestations may also occur in response to other micronutrients excess, as observed upon acute overexposure to the metalloid selenium [41]. As such, a tight regulation of brain iron homeostasis is of the utmost importance.

One of the processes for which the maintenance of iron homeostasis is vital is the regulation of axon myelination and, subsequently, neural transmission. In fact, a great number of studies present iron deficiency in early development as capable of disrupting synaptic function, with potential long-lasting effects that were shown to be associated with impaired cognitive and/or altered social behavior [26,42,43,44]. However, the importance of iron in the brain further relies on iron being part of the structure of many enzymes involved in energy metabolism and neurodevelopmental mechanisms [42,45]. In many of those, iron acts as a co-factor, which ensures proper functioning. This is the case for cytochrome c oxidase, which is a central enzyme for energy metabolism, tyrosine hydroxylase, which is responsible for dopamine synthesis, and for tryptophan hydroxylase, which is responsible for serotonin synthesis. Iron deficiency anemia has also been associated with decreased monoamine oxidase activity in humans, which promotes a disruption of the metabolism of monoamines, including dopamine and serotonin. In addition to iron’s role in the monoaminergic system, it further regulates the balance between inhibitory and excitatory neurotransmitters, γ-aminobutyric acid (GABA) and glutamate, respectively. Moreover, iron was shown to interact with other metals such as zinc, and to be involved in processes such as dendritogenesis, neuro-metabolism, gene profiles, and protein profiles [26,42,46]. 

Among all age populations, infants and young children are the most sensitive to variations in iron levels [20,47]. Presumably, this is associated with their rapid growth, which requires iron to prevent a decline in functions that are mainly exerted by the basal ganglia and that could lead to an impaired psychomotor development, as well as disrupted cognition and behavior [43]. Contrary to expectations, the effects of iron supplementation to children affected by iron deficiency are still controversial. These interventions were able to correct for iron deficiency and anemia, but their impacts on motor, cognitive, and behavioral outputs are ambiguous. This has been reasoned to result from methodological insufficiencies and heterogeneity of the study design [8,18,20]. These data will be thoroughly discussed under the section “Linking iron deficiency to cognition and social behavior in human subjects.”

While there is widespread acknowledgment of the highly deleterious effects of an increased iron status, particularly in the brain, the use of iron-enriched foods and beverages, used to overcome the negative impacts of iron deficiency, is widespread. However, the potential negative impacts of very high iron intake have not been established [48]. As such, several studies have started to address the effects of high iron intake or high peripheral iron status on neurophysiological mechanisms, cognition, and pathological development. While, in mice, there seems to be very strong indications that high iron intake in early post-natal life has adverse effects on cognition and neuropathological outcomes. The results of studies with human subjects are controversial. This is presumably due to methodological variations. Moreover, the effects of iron supplementation on cognition and pathological progression were only addressed in adults. While the findings are somewhat supportive, high iron intake in adults contributes to the degradation of neuronal function and pathophysiological development. No studies have addressed whether and how iron supplementation in early childhood impacts on adult cognition and behavior, as well as on brain aging and health [32]. Data addressing potential links between iron accumulation, cognition, and social behavior are either inconsistent or absent. Before discussing this topic in depth, we will focus on the details of iron metabolism and cytotoxicity, since these mechanisms are critical for understanding the processes involved in disruption of iron homeostasis and its potential impacts.

## 3. Iron Metabolism

Since the disruption of iron homeostasis has such severe consequences, the levels of systemic and intracellular iron need to be tightly regulated. In fact, many mechanisms contribute to maintain iron homeostasis, with a vast number of genes being responsible for the control of iron uptake, storage, and recycling. As for iron excretion, no physiological mechanisms have been described so far, with iron losses being presently described through menstruation and skin desquamation [49].

In the specific case of the brain, iron crosses the blood brain barrier (BBB), as iron-loaded transferrin (holotransferrin, HTf). Internalization of the complex iron-Tf:Tf receptors (TfR), expressed on the luminal side of brain capillary endothelial cells, allows its endocytosis. Subsequently, iron is released in acidic endosomes from Tf, and then released from the Tf-TfR complex. Once reduced to ferrous iron, it is translocated into the cytosol by the action of DMT-1. This iron contributes to the labile ferrous iron pool. However, poor expression of DMT-1 on brain capillary endothelial cells raised concerns about the requirement of this transporter for iron entry to the brain. In fact, alternative mechanisms indicated that iron could also be released from endosomal compartments via the expression of transient receptor potential mucolipin-1 (TRPML1) channels. Disrupted iron homeostasis observed in the brain of TRPML1-deficient mice further supports this notion. In addition, dysregulated iron metabolism has been observed in patients suffering from mucolipidosis type IV (ML4), which is a neurodegenerative disease in which a TRPML1 mutation is responsible for reduced brain ferric iron. This could lead to impaired myelination and neuronal damage [50,51]. Whether other iron transporters, like TRPML2 or ZIP8, can also play a role in regulating iron metabolism in brain capillary endothelial cells has not been fully elucidated.

Similarly to DMT-1, TRPML1 transports ferrous but not ferric iron. This suggests that a ferric reductase is required for TRPML1 to release iron from endosomal compartments. Whether this is ensured by one of the Steap family members remains to be established, but the ubiquitous brain expression of Steap 1 and high levels of Steap 2 render these proteins to be strong candidates for this function. Thus, the enzymatic activity of these proteins might increase iron uptake into the cells [52]. Additionally, stromal cell-derived receptor-2 (SDR2), which is a homologue of duodenal ferrireductase DcytB, is expressed in the brain, although its specific localization and function remain to be fully established [53].

Still the object of the discussion is the possibility of iron entering the brain via a transcytosis mechanism. This implies the exocytosis of the recycling endosome, which contains iron-Tf complexes. Their dissociation from TfR at the abluminal membrane results in the detachment of iron from Tf and subsequent release of Tf into the brain interstitium [54]. This potential mechanism of iron entry into the brain is supported by the reported normal amounts of iron in the brain of hypo-transferrinemic mice, i.e., animals presenting less than 1% of circulating Tf [55]. Regardless of the mechanisms, once inside the cells, iron is either directed to mitochondria, to participate in processes ensuring cellular function, or is stored in the cytosol, within the multimeric ferritin L and H chain subunits [33,56]. Excess iron is then exported to the extracellular space by ferroportin, which is the only iron exporter that has been identified so far [57]. The expression of ferroportin on the abluminal membrane of brain capillary endothelial cells is coupled with ceruloplasmin, which presents a ferroxidase activity capable of re-oxidizing ferrous iron into ferric iron and allowing it to enter the Tf distribution cycle. This has led the scientific community to hypothesize that endothelial cells regulate iron entry into the brain by acting as gate-keepers for iron and releasing it when required.

Most importantly, levels of circulating Tf in the brain interstitium are quite low, which implies Tf to become saturated with small amounts of iron. This justifies non-Tf bound iron (NTBI) to be the main source of iron delivery to neural cells. NTBI might have entered neural cells by binding to DMT-1 and should be, subsequently, redistributed to the extracellular space in the brain parenchyma. High levels of ferrous iron can also be found in the cerebrospinal fluid [58], and possibly represent the biological underpinning of the existence of multiple mechanisms guaranteeing iron homeostasis. NTBI can also be up-taken by neurons and enter these cells via DMT-1, Zip-14, or other molecules ensuring non-vesicular import mechanisms [59]. Ferritin can also bind NTBI and cross the BBB via a transcellular transport through the T-cell immunoglobulin and mucin domain-containing 1 receptor 1 (TIM-1) [60], which is the human homologous of the rodent gene TIM-2. This mechanism ensures endocytic iron reutilization in oligodendrocytes [61]. Moreover, the relevance of ferritin as a possible route for iron delivery to the brain was also shown in microglia. These cells can act as an exogenous iron source considering their ability to increase ferritin expression when attempting to limit the amount of extracellular-free iron in the brain. This means that the upregulation of ferritin in microglia is a direct consequent of NTBI uptake [62].

Fenestrated capillaries of the choroid plexus are also playing a dominant role in regulating iron entry into the brain. Their peculiar permeability facilitates not only the diffusion of molecules of different sizes to which iron can bind, but also their regulated entry into the brain through the ferroportin-hepcidin axis present in these cells [63]. In the brain, iron can be transported as described or bound to low-molecular-weight agents such as citrate, ascorbate, or ATP [4]. While ATP and other nucleotides may contribute to the release of iron via the exocytosis-dependent abluminal membrane transport [64], the higher concentrations of citrate and ascorbate when compared to their concentrations in circulation could be justified by the need to prevent NTBI from dysregulating iron homeostasis. Although the affinity of these molecules for iron is lower than that of Tf, their abundance allows to bind significant amounts of NTBI.

Another major player in iron metabolism is hepcidin, which is the iron regulatory hormone. Contrarily to hepcidin role in the peripheral compartment, its role in the brain is still under debate. Currently, it is known that hepcidin targets ferroportin in the brain. However, growing evidence supports the ability of systemic hepcidin to cross the BBB and enter the brain. On the other hand, the existence of an autonomous mechanism justifies the absence of brain iron accumulation in mice or patients presenting hepcidin mutations, such as in hemochromatosis. Additional mechanisms can then be responsible for the maintenance of brain iron homeostasis. These mechanisms should involve microglia and astrocytes, which are the brain cells producing higher levels of hepcidin [65].

Despite the numerous mechanisms regulating iron homeostasis in the brain, this tight regulation can still be disrupted. The following section addresses these mechanisms and the impacts of their disruption.

## 4. The Cytotoxicity of Iron in the Brain

The main causes underlying brain iron accumulation still need to be studied in more detail. Nonetheless, and in addition to other types of brain iron deposits and superficial siderosis, the literature points at cerebral microbleeds as one of the major factors involved in brain iron overload [66]. These microbleeds are brain microhemorrhages that can occur physiologically in the elderly and affect both brain tissue and ventricles. Microbleeds are often asymptomatic and a recurrent headache is the major complaint. However, they are not always silent. This is the case upon release of hemoglobin into intracranial cavities, which can lead to clinical manifestations such as weakness, vomiting, seizures, decreased level of consciousness, neck stiffness, and others [67].

When unlocked from cells, hemoglobin is oxidized and rapidly broken-down to its by-products, heme, and iron [2]. Therefore, depending on the extent of cerebral microbleed, brain iron accumulation can compromise synaptic transmission and neuronal functions [68]. This suggests that individuals that are more susceptible to vasculature impairments, such as patients affected by genetic blood disorders like sickle cell or thalassemia, could present higher risk of cerebral microbleeds. Those microhemorrhages could contribute to neurological conditions that these subjects might suffer from [69]. In fact, this is supported by a study with children affected by hemoglobinopathies [70], and developing a reversible neurological complication, known as leukoencephalopathy syndrome, upon blood transfusion [71]. This means that blood treatments might cause long-term effects to organs such as the brain, which might result from potential iron accumulation over time. Still, iron toxicity is prevented by storing excess iron in ferritin and intracellular vacuolar-like structures, like lysosomes. Therefore, this does not come as a surprise regarding the importance attributed to lysosomal impairment during aging [72]. The role of lysosomes in ferritin degradation [73] allows us to understand their strict relationship with brain pathologies [74] like neurodegenerative diseases such as Alzheimer’s disease [75,76]. These disorders have been defined as iron storage diseases, pathologies in which iron accumulates as encapsulated iron, i.e., iron contained within vesicles [77,78]. Currently, it remains to be elucidated whether this iron is toxic or accumulates in response to chronic stress, acting as a stress responsive intracellular second messenger, as recently proposed [79]. It is, therefore, possible that iron becomes toxic only after certain thresholds. All these questions will remain opened until final results of iron chelation therapy on neurodegenerative diseases, such as Alzheimer’s and Parkinson’s disease, are fully disclosed. Nevertheless, the reduction of iron accumulation in patients with Parkinson’s disease and treated with deferiprone was considered satisfactory [80,81]. New compounds, like the chiral 3-hydroxypyrid-4-one derivative, which present the same properties but prevent the side effects of neutropenia and agranulocytosis after prolonged exposure to iron chelation treatment, are currently being tested in different models of Parkinson’s disease models.

Despite the numerous mechanisms to prevent iron cytotoxity, the ability of iron to transfer electrons allows its participation in the Fenton chemistry and the generation of highly reactive and harmful hydroxyl radicals (HO•), from the conversion of diffusible H_2_O_2_ [82]. Once produced, reactive oxygen species (ROS) can damage lipids, proteins, and nucleic acids, which leads, ultimately, to cell death. In the specific case of the brain, the high abundance of lipids renders it very susceptible to oxidative stress. Since these lipids are ensuring neuronal communication by mediating impulse transmission, brain oxidative stress is considered among the etiology of most brain disorders [34,83]. In addition, the irreversible damage caused by iron fueling ROS production in the brain is also due to the lower tolerability and repair capacity of this organ when compared to other tissues [84].

Therapeutic interventions based on antioxidant administration have not been successful to prevent the neural toxicity of iron-driven oxidative stress. The inefficiency of drugs to cross the BBB could justify the lack of positive outcomes. However, this could also be influenced by the need of a small amount of ROS production for cell metabolism, which these drugs are suppressing. For example, microglia produce ROS to activate phagocytosis and eliminate possible harmful stimuli in the brain parenchyma. ROS further regulate the activity of both kinases and phosphatases, and modulate signaling pathways activated by transcription factors. In neurons, most ROS are derived by oxygen consumption, as by-products of mitochondria energy production [85]. The abundance and intense activity of these organelles in the brain, as they provide the energy for daily activities, renders the brain highly susceptible to oxidative stress. For example, meta-analytic studies have shown that increases in oxidative stress are associated with depressive disorders, including major depressive disorder and bipolar disorder [86]. This notion is also supported by the fact that mitochondria are the main source of intracellular iron [87]. A link between chronic exposure to stressors, maladaptive mitochondrial changes, and neurodegenerative disorders like dementia and AD, has also been suggested. Both heme synthesis and assembly of iron-sulphur clusters occur in the mitochondria, where density and activity vary, according to brain regions. This explains the existence of areas that are more sensitive to iron-driven oxidative damage than others. As an example, the substantia nigra and the striatum are areas where iron mostly accumulates and reaches higher concentrations when compared to the hippocampus, cortex, pons, and medulla. These latter regions present the lowest amount of iron [33]. The oxidation of dopamine to neurotoxic metabolites, which occurs in the substantia nigra and is coupled to increased iron levels, further boosts oxidative damage and justifies the sensitivity of this region to neuronal death [88]. As mentioned above, the main target of ROS production in the brain is neuronal membranes rich in polyunsaturated fatty acids (PUFA). Lipid peroxidation is a key feature defining iron-mediated cell death, also known as ferroptosis [34,89]. Still, iron’s role in impairing brain activity, cognitive functions, neuroplasticity synaptogenesis, and synaptic transmission remains to be elucidated. Nevertheless, epidemiological studies with PUFAs supplementation were shown to reduce the risk of neurological and psychosocial disorders [90]. Thus, a better understanding of causes leading to iron-driven oxidative stress is important for the development of effective therapies that prevent neuronal damage and functional impairment. The following section will precisely start to address the potential links between the neural circuits involved in iron regulation, cognition, and social behavior.

## 5. Neural Circuits and Social Behavior

Social behavior is one of the main areas of current research in fields as diverse as anthropology, sociology, psychology, or neuroscience. Simply put, social behavior is how we do things in social contexts. If one focuses on human social behavior, we are talking, for instance, about how we perform our jobs or how we spend our holidays, how we discuss things with others, or how we run away from them. Any of the above-mentioned behaviors are framed by knowledge and dispositions that we have embodied through our life trajectories, perceptions of the situation that we are being confronted with, and its broader social-economic contexts, our motivations, and expectations, as well as biological factors, mechanisms, and the more general physiological status. Changes in these processes characterize many neuropsychiatric disorders and psychosocial dysfunctions. This justifies an increasing interest in understanding the molecular mechanisms regulating neural circuits involved in the development of social behaviors. Since most genes are conserved across species, once again, research in rodents was fundamental to dissect specific brain networks involved in the development of social behavior. In human subjects, the brain structures involved in cognitive processes were originally identified through magnetic resonance imaging (MRI) studies, which revealed activation of specific regions upon the development of specific social tasks [91]. The somatosensory and temporal cortex are involved in perception and recognition of facial emotions [92]. The amygdala is involved in fear processing and threat detection [93] and the prefrontal cortex is responsible for decision-making and behavior, as well as performing executive functions [94]. Changes in the amygdala response can result in social anxiety disorders, and damages to the frontal cortex can underlie psychopathological and antisocial behavior [95]. Still, the existence of neural circuits interlinking specialized cortical and sub-cortical structures ensure that these areas work together to ensure that a certain behavior takes place [91].

From a molecular point of view, a series of neurotransmitters and neuromodulators regulate the activity and maturation of these brain areas. Although glutamate, GABA, serotonin, and noradrenaline contribute to modulate pre-frontal-striatal circuits, among neurotransmitters, dopamine stands out. The involvement of dopaminergic projections is responsible to exert functions resulting from the cooperation of all previously described areas for sustained attention, memory storage and retrieval, emotion regulation, and motivation. Since iron impairs dopamine synthesis, by influencing the activity of the enzyme that produces it, i.e., tyrosine hydroxylase, and fosters the production of ROS through dopamine catabolism [96], others have suggested that the outcomes of neurocognitive tasks are highly influenced by any disturbance in iron homeostasis [47]. It is precisely the link between brain iron homeostasis, cognition, and social behavior that will be addressed in the following sections of this article. Special attention will be devoted to studies with human subjects.

## 6. Linking Iron Deficiency to Cognition and Social Behavior in Human Subjects

**Figure 3:**
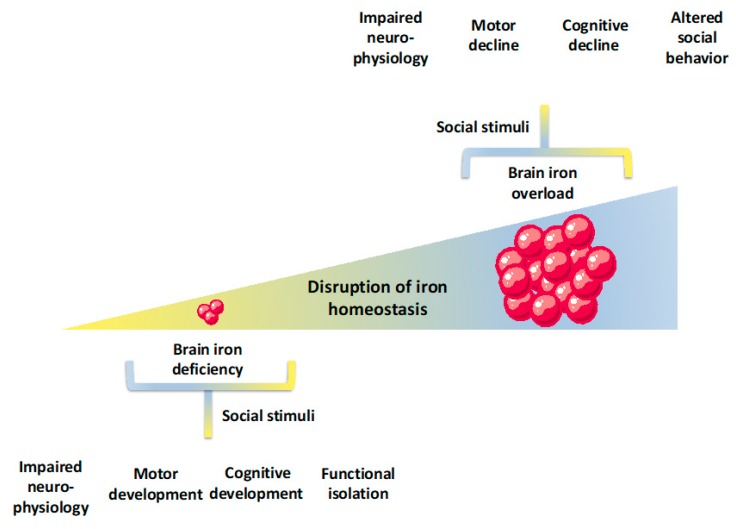
Multilevel repercussions of iron deficiency and iron overload in the brain. Brain iron deficiency is associated with disruption of neurophysiological mechanisms that, within a social context that does not provide regular stimuli, compromises motor and cognitive development (e.g., impaired motor sequencing and bi-manual coordination, poor executive function, attention, and memory). Subsequently, these can impact social behavior, which leads to, for instance, functional isolation. As for the impaired neurophysiological mechanisms associated with brain iron overload (e.g., exacerbated brain oxidative stress, neuronal cell death), these are associated with motor and cognitive declines (e.g., motor slowing, insufficient adjustment and altered feedback processing and sensitivity, memory loss, and impaired decision-making). Within a social context that does not provide suitable stimuli, these might impact social behavior leading to, for instance, hesitant behavior and unwillingness to interact with others.

Many studies have shown that iron deficiency with or without anemia is associated with short-term and long-term impacts on psychomotor development, cognition, and social behavior in human subjects (Figure 3). While iron deficiency is more prevalent and studied in early childhood, which is a period of rapid growth and high needs for iron, an elevated risk for iron deficiency is also present in women of reproductive age, as a result of regular blood loss during menstrual cycles, and during pregnancy, which is characterized by increased iron requirements [8]. In fact, some of the previously mentioned neurophysiological, cognitive, and behavioral features of early iron deficiency were also reported to be present in iron deficient women of reproductive age [97,98,99]. During pregnancy, iron deficiency anemia has been associated with prematurity, low birthweight neonates, maternal morbidity, and impaired mental and psychomotor development [4,8,100]. Even though the number of studies is presently limited, future studies should address whether these modifications are the strict result of iron deficiency or, rather, of its interactions with other factors.

In what concerns the mechanisms connecting a disruption of iron status to neurophysiological and cognitive impairments and alterations in social behaviors, it has been previously suggested that an iron-induced disruption of major dopamine pathways (i.e., mesocortical, mesolimbic, nigrostriatal, and tuberohypophyseal), would possibly impair its ability to propagate neuronal impulses. This disruption could potentially lead to motor and cognitive impairments, altered social behavior, and/or pathological development [47]. A different line of research has suggested that iron deficiency can be deleterious to axon myelination. In fact, it has been documented that early iron deficiency is associated with short-term and long-term latency delays in visual and auditory evoked potential studies. The latest study results that are consistent with impaired axon myelination have lower amplitude in the variability of heart rate in the sleep-wake cycle [101,102,103]. This could be due to the documented alterations in iron status, but also due to its impacts on different neurotransmitters such as serotonin, norephinephrine, or others. It is also possible to find some studies in which the evoked potential analysis was inconclusive and the results were attributed to problems in the study design [104].

Specifically, regarding the dopamine-dependent pathways, studies have shown that children and young adults with chronic, severe iron deficiency with or without anemia in infancy, present altered dopamine frontal-striatal circuits, known to be involved in the control of executive functions, sustained attention, memory, emotion regulation, and motivation. In particular, the authors show that early severe iron deficiency was most likely contributing to impairments in neurocognitive and motor functioning and modifications in social behavioral in infancy, at 5 years of age and at 11–14 years of age. At the age of 19, cognitive impairment, as revealed by poor inhibitory control and executive functioning, was shown to be present [47,103].

The negative impacts of iron unbalance on dopamine action can also affect the mesolimbic pathway, which connects the ventral tegmental area in the midbrain to the ventral striatum of the basal ganglia in the forebrain. Alterations in this region, where dopamine plays a central role in behavioral activation, inhibition, positive affect, and reward [105,106], were proposed to be involved in increased prevalence of wariness and hesitance behaviors, lack of positive affect, diminished response to novelty, and social engagement. These modifications, present in infants with iron deficiency, with or without anemia, seem to show a dose response to the severity of the iron deficiency [43,107,108,109,110,111,112,113,114].

The dopamine nigrostriatal pathway is involved in movement control and regulation. In fact, infants with iron deficiency with or without anemia were shown to have impaired motor sequencing and bi-manual coordination, which is a symptom that was more severe in anemic infants [115,116]. In a different study, iron deficient infants, with or without anemia, presented a lower rate of spontaneous eye blink, which is a widely accepted measure of the nigrostriatal pathway (anemic infants had the lowest levels). The study further showed that a spontaneous eye blink could be partly corrected with iron supplementation in anemic infants [117]. Children with iron deficiency also present a disrupted learning process, which results in impaired cognition and altered social behavior. 

Lastly, the involvement of the dopaminergic tuberohypophyseal pathway has been studied fewer times. Nevertheless, dopamine production by the hypothalamus inhibits prolactin release by the anterior pituitary. As such, decreased dopamine function as present in iron deficiency should be associated with increased prolactin levels. This was shown to be the case in iron deficiency anemia in infancy [118]. Even though these results are indicative of an impaired tuberohypophyseal pathway and long-term dysregulation of prolactin, one should note that the regulation of prolactin is complex and involves other neurotransmitters such as serotonin.

At this point, there are several important arguments to put forward. First, the brain works as an integrated system and, as such, responses are unlikely the result of impairments in the action of specific neurotransmitters, regions, circuits or processes, but rather result from the concerted action of interconnected and interdependent neurotransmitters, processes, circuits, and regions. Second, other dimensions could have a role in mediating or potentiating the biological and neurophysiological impacts of iron deficiency. In this line of reasoning, a very important dimension to consider when analyzing the impacts of iron deficiency in cognition and social behavior in human subjects was not yet addressed in this section. What is the role, if any, of the social dimension? Social psychology and sociology have shown that the social context in which individuals are embedded influences individual cognition and action. However, there is little, if any, evidence of how it acts concertedly with the neurophysiological impacts of iron deficiency.

In fact, some studies have started to give innovative inputs into this discussion. In 2013, Algarín and colleagues developed a study with 10-year-old children who had early iron deficiency anemia and were given iron supplementation for at least six months [103]. At 10, these children presented hemoglobin levels within a normal range but still had impaired executive functions, as assessed by slower reaction times, less accuracy, and poorer inhibitory control as compared with children that did not present early iron deficiency anemia. Since the iron deficiency anemia and the control groups were matched for a socio-economic status, Algarín and colleagues discarded the potential effects of the social context. In line with the “critical period hypothesis,” stating that an absence of adequate nutrients in critical developmental periods results in permanent structural deficits that cannot be corrected with nutrient supplementation after that period [119], the authors argue that these results are consistent with long-term effects of iron deficiency anemia on axon myelination and dopamine-dependent prefrontal-striatal circuits. These results could also be consistent with the “Altered-regulation hypothesis,” which states that early nutrient deficiency modifies the brain epigenetic landscape via CpG methylation, histone modification, or hydroxymethylation [120].

In a different study, Carter and colleagues have shown that nine-month old infants with iron deficiency anemia presented poorer attention and recognition memory than infants without anemia (both with and without iron deficiency) [121]. These effects were at least partially mediated by a decreased ability to engage affectively with the environment and by other socio-emotional modifications. The authors further show that there was no relation between the impairment of cognitive function and maternal socio-demographics. These data raise questions about the role of the social dimension in the cognitive and behavioral factors that characterized these children, which shows that it probably involves other factors beyond socio-demographic characteristics.

However, one could easily argue that cognition and behavior are framed by a multitude of psychological and social dimensions (including norms, beliefs, knowledge and dispositions, interaction context, actors’ perceptions, appraisals, expectations and motivations, among others) [122,123]. To give a specific example, one of these processes is known as personal reflexivity, which is the capacity that individuals have to think about themselves and the world, their decisions and interactions, as well as to project future behavior [124,125,126]. The authors have found diverse profiles of personal reflexivity, framed, among others, by the individuals’ social origins. The social–economic contexts in which they live, their life trajectories and interactions, as well as the available resources. Most importantly, these profiles of individual reflexivity impacted not only how individuals think about themselves and the world, but also how they planned for their future and acted in social contexts. As such, cognition and behavior cannot be subsumed to a one-to-one linear relation either with the social-economic status or with the maternal socio-demographics. In fact, an immense number of studies have shown that the development of executive functions and subsequent social behavior, are also highly dependent on the stimulation cues that one receives from the social environment throughout their lives. These cues characterize social interactions in general (i.e., who you interact with, what type of interactions you have, how frequently you have them and others), as well as interactions during formal learning processes [127]. More specifically, it has been consistently shown that environmental interventions can potentiate cognitive functions, such as working memory [128,129,130]. If this is the case, an analysis of the social-economic status, or of the maternal socio-demographics, in spite of providing most relevant inputs, is not able to capture the complexity of the social environment and the individuals’ appraisal of that same environment. Given the above, even though these studies do present clear associations between early iron deficiency anemia and cognition, they definitely do not allow us to rule out the role of the social dimension in cognition.

In fact, a couple of studies already shed some light into this discussion. One study shows that five-year-old children with early iron deficiency anemia are less active, more inhibited, and shy in a mother-child interaction [131]. These behavioral patterns are similar to the ones presented by other malnourished children [132,133]. Concomitantly, the same study reports that the mothers of children with normal iron status are more responsive and have richer interactions with their children. As such, the modifications that children present in social behavior could result from the combined effect of early iron deficiency anemia and decreased social interaction patterns that these children were subjected to. Adding on to this line of reasoning, more recent studies have confirmed children with iron deficiency to be less active, emotionally dull, and disengaged [112]. In addition, iron-deficient children seek and receive less stimulation from caregivers, which is a process described as “functional isolation” [134,135,136]. The authors argue that, over time, these behaviors result in smaller motor, cognitive, and social inputs from the environment and could interfere with social learning processes and development. To address this issue, it has been proposed that interventions for iron-deficient children should target social interactions [135]. In fact, this strategy was already mobilized on a study combining iron supplementation with a home intervention, designed to foster child development by providing support to the mother-infant relationship and has shown some positive effects on cognition and behavior [137]. Other authors have suggested that iron supplementation should be accompanied with cognitive training [138], such as working memory training, shown to present positive outcomes in cognitive functions [127].

Altogether, this clearly points to a role of iron deficiency and psychological and social processes in the development of cognition and social behaviors. The point being made here is that, despite the relevance of iron and of its neurophysiological action, these mechanisms are most likely interdependent with psychological and social dimensions, as individuals live in social contexts, grow in social contexts, learn in social contexts, and construct their memories in social contexts. Future studies should provide an in-depth characterization of all these dimensions, as well as of the specific interconnections among them.

## 7. Increased Iron Levels, Cognition, and Social Behavior in Human Subjects

The link between cognitive impairment, modifications of social behavior, and brain iron accumulation in human subjects is not well-understood. The few studies focusing on the impacts of elevated iron levels in human subjects are characterized by wide differences in research design and socio-demographic characteristics of the analyzed population. Furthermore, wide variations on population size and outcome measures render available data to present inconsistencies that are difficult to reconcile [32]. Additionally, unavailability is a characterization of social behaviors and social contexts of the studied population, which is a characterization that, as previously pointed out, is of major relevance when studying cognitive processes. In addition, while it would be of major relevance to understand whether high iron intake in the early childhood period, a common supplementation for neonate/infant iron deficiency, impacts brain mechanisms and cognition in adult life, no studies have addressed this issue. These studies are necessarily timely and costly, but, considering the increasing life expectancy, prevalence of neurodegenerative diseases as well as the relatively widespread intake of iron-enriched food and beverages to prevent or overcome early iron deficiency, these studies are of the utmost relevance [32].

Nevertheless, previous scientific studies still present some hints that should be taken with careful consideration when discussing potential impacts of iron overload in cognition and social behavior (Figure 3). Studies with human subjects have mostly focused on whether high iron intake or high peripheral iron in adults were associated with poorer cognitive outcomes (we will address the impacts of brain iron accumulation in psychosocial disorders in the following sections of this paper). In this line of research, some studies have shown no associations between plasma or serum iron levels and cognitive outcomes [139,140,141] (one cross sectional study with 2000 subjects with more than 65 years old and two cohort studies with approximately 800 subjects with ages above 50 years). Still, other studies present the opposite outcome, which reveals an association between diverse measures of iron status and cognition. More specifically, a cross sectional study involving 1451 subjects (mean age of 75 years old) revealed an association between plasma iron levels and cognition [142]. On their hand, Umer and colleagues have shown that mild cognitive impairment is present upon high serum iron (98%), ferritin (56%), and transferrin saturation (107%). This was a cross sectional study, developed on 87 nursing home residents with more than 65 years old and who presented no neurologic diagnosis [143]. In 2012, Mueller and colleagues have shown, in a relatively small study, that the progression from mild cognitive impairment to Alzheimer’s disease was associated with serum copper to non-heme iron ratio (19 control subjects, 11 cases of stable mild cognitive impairment, 7 cases of progressive cognitive impairment, and 19 cases of early dementia, mean age of 78 years old). Lastly, pre-menopausal and peri-menopausal women presented an association between improved cognitive outcomes and lower serum ferritin levels (n = 4959 men and women, 35–60 years old) [144]. Taken together, the data gathered with human subjects with results obtained with animal models, which show a clear association between increased iron and cognitive impairment [32], there seems to be some indications for a link between systemic iron status and cognition that needs to be studied in more detail.

A diverse approach further addressed iron’s impacts on cognitive decline in older populations. These studies focused on the association between cognitive function and brain iron deposits, which are shown to be present in several brain regions. The presence of these deposits has been mainly attributed to impairments in the regulation of brain iron [145], brain microbleeds, focal micro-hemorrhages, with iron deposits in lobar white matter, basal ganglia, and internal capsule [67], and superficial siderosis, which are accumulations of hemosiderin in subapial layers of the brain [146]. These studies have reported that increased iron deposit volume was associated with decreased cognitive ability [147,148,149,150,151,152,153]. However, the association of aging, iron deposits, and impaired cognition remains to be clarified. While one extant cross-sectional study presented data consistent with such a notion [150], the only longitudinal approach to the role of iron aging-associated cognitive decline failed to confirm the previous results [151]. Despite the divergences in the scientific literature, since the reported patterns of iron deposition in the brain of healthy human subjects were similar to the ones present in neurodegenerative diseases, it has been proposed that iron deposits could be used as an indicator of early prediction and diagnosis of neuropathological progression [152].

There is yet a different line of studies focusing on the relation between increased iron levels, neuronal mechanisms, cognition, and social behavior, which could reveal some interesting cues. Studies focusing on iron-induced oxidative stress [86] suggest that iron might play a key role in reactions to psychosocial stress, which impact neuronal mechanisms and cognition. In fact, it has been shown that psychosocial stress, which was previously associated with oxidative stress in animal models and human subjects [154,155], can lead to iron accumulation in the brain, which exacerbates brain oxidative stress and leads to a neuronal cell in rats [156]. This mechanism is aggravated by iron overload [157], which is a condition with increasing prevalence in healthy men, postmenopausal women, and the elderly population and has been attributed to changes in dietary habits [158,159]. These data suggest a reinforcement mechanism. Not only stress can disrupt brain iron homeostasis, contributing to iron accumulation in the brain, but also increased iron uptake further impairs brain oxidative balance and, subsequently, brain functioning. Taking in account the increasing age of the world’s population and changes in diet, it would be particularly relevant to have a more complete understanding of the mechanisms mediating stress-induced iron accumulation, iron overload, and brain functioning.

In order to have a full sketch of this system, one should look further at the relation between psychosocial stress and cognition. In fact, it is known that decision-making, the process of choosing between alternative courses of action, can become impaired under stressful conditions [160]. Decision-making processes develop via neurophysiological mechanisms and are framed by broad social-cultural contexts, perceptions, and appraisal of the situation, available knowledge, decision content, motivations, and expectations [161,162,163]. Furthermore, it was also shown that psychosocial stress activates internal biological processes favoring the occurrence of an inflammatory response [164,165,166]. This strict correlation between stress and inflammation is detectable even in healthy individuals but overwhelmed with time constraints and increased task demand [167,168]. Upon prolonged stressful conditions, neuronal activity, motivation, and goal-directed motor behavior start to decrease, which is an effect that is dependent on increased levels of pro-inflammatory cytokines [169]. Lastly, these changes in brain function result in anhedonia, fatigue, motor slowing, psychomotor retardation, and isolation in mice [170]. In human subjects, stress promotes the mobilization of dysfunctional strategies such as looking for premature closure and non-systematic scanning, insufficient adjustment, and altered feedback processing and sensitivity [160]. Additionally, the association between stress and decision-making was shown to be shaped by several social moderators, such as the nature of the stressor, the individual stress reaction, or individual characteristics [171]. Research also shows that the appraisal of the stressor itself, either as a challenge (i.e., the situation is seen as an opportunity for mastery or personal growth) or as a threat (i.e., when demands are seen as exceeding the available resources), should be taken into account, since it influences performance [172]. As such, we know that brain iron accumulation is present under conditions of psychosocial stress. We also know that psychosocial stress impacts on cognitive processes such as decision-making as well as psychological and social dimensions are critical for decision-making processes in the presence or absence of stressful conditions. It remains to be ascertained whether and how iron accumulation plays a role in these processes, directly or indirectly contributing to compromising cognitive function, including impaired appraisal of the environment and decision-making processes. Some hints can be given by looking closely at studies focusing on chronic social defeat stress (CSDS), which is a neuropathology that we will focus on in the following section.

Lastly, as for iron deficiency, it is our understanding that future studies should definitely consider an integrated analysis of iron neurophysiological mechanisms, cognitive processes, and thoroughly characterize social behaviors and its contexts. This is the case since it is known that cognition and behavior are also dependent on the stimulation cues one is subjected to from the social context in which we live in [128,129,130] as well as by broader social-cultural structures, interaction contexts, actors’ perceptions, dispositions to act, motivations, and expectations [162,163]. These social cues, which are expected to act concertedly with the biological mechanisms, could interfere with the biological footprints of an iron dysregulation on cognition and behavior. This renders it difficult to understand the mechanisms at stake.

## 8. Linking Iron with Chronic Stress, Anxiety, and Depression

Social behavior and stress are deeply connected, and this interrelation was firstly revealed by experiments conducted in animal models describing how social interactions in rodents can cause stress and how social behavior can change in response to environmental stressors [173]. In human subjects, stress caused by social interactions was shown to profoundly impact individuals’ health and well-being [174]. In fact, both biological and neurobiological mechanisms underlying social behavior are influenced by molecular changes characterizing stress, which are understood as the physiological or psychological response to a prolonged internal or external stressful event (i.e., a stressor), even when it is no longer physically present but is recollected by the individual [175]. When stress is prolonged, the constant state of alertness that individuals are subjected to results in the development of chronic stress, which is associated with chronic fatigue and characterized by low levels of vitality, vigor, and energy [176]. Chronic stress leads to worse cognitive performance and impairs decision-making, including information processing, learning, and risk-taking [177].

Chronic stress has a major impact on the human brain, which alters the structure, connectivity, and neurochemistry of affected areas. Profound changes are mainly observed in hippocampal morphology, function, and neurogenesis [178], which are then associated with a neuroinflammatory profile and impaired neuronal plasticity. Dysfunction in neuronal connections of the basolateral amygdala can be triggered by stress, and compromise the structure, connectivity, and density of excitatory and inhibitory neurons of this region [179]. The release of molecules known as stress mediators and their binding to correspondent receptors activates biological mechanisms, which allows a subsequent adaptation to the challenging situation and shifts the physiological balance toward a new homeostasis. In this phase, oxidative damage and cognitive decline were pointed out as capable of modulating the response to chronic stress and the brain’s ability to fight against it [180].

From a molecular point of view, exposure to chronic stress activates a neuroendocrine response, which results in the production of the stress hormone, cortisol, and in its subsequent release into circulation. Although there is little research on the ability of iron to modulate the neuroendocrine system in human subjects, lower cortisol levels were shown to be produced in conditions of serum iron deficiency [181]. The correlation between cortisol and iron could be justified by the notion that the enzyme necessary for the synthesis of this stress hormone is an iron-containing protein. Hence, cortisol synthesis strictly depends on iron levels [182].

The autonomic nervous system and endocrine signals involved in cortisol modulation of neural and behavioral responses are influenced by the circadian rhythm, which, in turn, is highly dependent on the levels of circulating iron [183]. More specifically, while cortisol levels are low around midnight, rise approximately three hours after and reach their peak in the morning [184], the opposite occurs for serum iron. Assessed as transferrin saturation, iron is higher at night and starts to gradually decrease during the morning. No circadian variations have been detected for transferrin receptors or ferritin expression [185]. The impact of iron on the circadian regulation of cortisol is due to most clock-related genes being iron-containing molecules [186], so one can extrapolate the effects of iron deficiency or iron accumulation for the production of this hormone. Since iron and stress were shown to have strong impacts on circadian rhythms, one can speculate that variations in this mechanism could be the basis of modifications in social behavior, and, eventually, a decline in health and well-being.

Disruption of the circadian clock is also associated with anxiety, which can be conceptualized as the response to stress and dangerous situations, and is present in more than 12% of the entire population. Anxiety is described by the current Diagnostic and Statistical Manual (DSM-5), published by the American Psychiatric Association (APA), as “characterized by feelings of tension, worried thoughts, and physical changes like increased blood pressure.” Thus, under the definition of anxiety disorders, the APA refers to those that share “features of excessive fear and anxiety and related behavioral disturbances” [187]. Anxiety is characterized by a high arousal negative state [188]. In the later decades of the twentieth century, its prevalence and consistent increase led researchers to claim that we live in “the age of anxiety” [189]. A number of different factors can trigger anxiety, and these were shown to be modulated by iron levels. As the first example, the more intuitive, anxious feelings are associated with iron deficiency, since low iron levels translate into a lack of oxygen to tissues. This means that this anxiety state can be reverted as soon as normal iron levels are restored [190]. The negative influence of iron on dopamine synthesis, as previously described, also contributes to develop a state of anxiety, given the importance of this neurotransmitter in maintaining attention and focus. While conditions of iron deficiency compromise the activity of tyrosine hydroxylase and results in lower dopamine production, the same occurs with excess iron, since this metal causes dopaminergic neurodegeneration by exacerbating the oxidative stress produced by dopamine degradation. Consequently, individuals suffer from cognitive changes, altered performance, and memory loss. In mice, the development of anxiety is likely accompanied with the accumulation of iron in the brain, which occurs upon a reduced expression of antioxidant enzymes and oxidative-mediated damage to catecholaminergic innervated regions of the anterior forebrain [191]. In rodents, anxious behavior can be improved by restoring the redox balance in these areas through the uptake of dietary or pharmacological antioxidants. Whether this occurs in human subjects needs to be fully elucidated. However, the consumption of fish-derived omega-3 poly-unsaturated fatty acids as well as docosahexaenoic and eicosapentaenoic acid aids preserve cognitive capacities via a mechanism that can possibly reduce iron-driven oxidative damage and restore the expression of antioxidant enzymes [90]. This further points at the need to monitor iron levels in psychosocial disorders, since the development of new interventions maintaining iron homeostasis could contribute to better outcomes.

The effects of iron deficiency on psychosocial disorders were further reported in depression. Several studies indicate anemia as a pre-disposing factor [192,193] for a condition that, unlike anxiety, is characterized by a low arousal negative state accompanied by pervasive negative thoughts and emotions about oneself and the future [188]. Individuals with major depressive disorder have difficulties engaging in cognitive reappraisal (i.e., an effort to change the subjective evaluation of a situation in order to modify its emotional impact), which is a key emotional regulation strategy to deal with stressful or uncontrollable events [194]. Early adversity, conflict occurrences, maternal depression, socioeconomic status, and others, can be considered as major causes for depression, and have been associated with low-grade inflammation. This is corroborated by prospective, longitudinal, and meta-analyses research. These studies show depression to be correlated to an increased immune response. Inflammatory cytokines, such as tumor necrosis factor (TNF), interleukin (IL)-6, C-reactive protein, the hemoglobin scavenger protein, and haptoglobin, have been found in the peripheral blood of patients suffering from severe depressive symptoms [195,196]. Thus, given the cross-talk between iron metabolism and inflammation [197], one can understand the involvement of genes controlling iron hemostasis in the pathogenesis of this disorder. 

Studies in mice show that stress-induced depression causes brain iron overload and affects specific regions of this organ, such as the hippocampus. Administration of iron chelation therapy to these animals prevents neuronal loss [198], which is an effect that is also observed in human subjects with depression when subjected to a similar treatment. Widespread iron deposition was found in the brain of these individuals and was recognized as capable of predicting both depression and memory impairment [199].

Thus, although the biological basis of chronic stress, anxiety, and depression remains very elusive [26,31,191,200,201], overall, iron is considered to be a key biological marker for psychosocial disorders associated with impaired cognitive functions and altered social behavior. As previously discussed, the potential effectiveness of iron-dependent treatment strategies should take into consideration that, even though cognition and behavior do develop through iron-dependent physiological and neurophysiological mechanisms, they are unequivocally framed by psychological and social processes that should also be addressed in future research.

## 9. Discussion

Dysregulation of iron levels in human subjects is a relatively common phenomenon. On the one hand, we have iron deficiency, which is the most prevalent nutrient deficiency worldwide, and, on the other hand, iron overload, which is common in the increasing aging population as well as in pathologies such as neurodegenerative diseases and psychosocial disorders. For both cases, the impacts of iron dysregulation in neurophysiological structures and mechanisms have been studied, with consistent data published in both animal models and human subjects. The association between iron dysfunction and motor and cognitive impairment, as well as modified social behavior, is also very consistent. However, while most studies show that iron interventions could correct for altered iron levels, this type of intervention was mostly unable to revert cognitive or behavioral modifications. Within the iron community, many authors either underline the methodological insufficiencies of intervention studies or argue that iron dysregulation during critical periods, cannot be reverted. Other authors argue that correction of iron levels necessarily needs to be complemented with cognitive stimuli and other types of social interventions, which is a proposal that is completely in line with the findings of social psychology and sociology.

It is our understanding that, while biological impacts or iron dysregulation are undeniable and deserve to be studied and discussed in great detail, its associated cognitive and social blueprints could be further exacerbated by psychosocial contexts that do not promote regular cognitive function and normative social behavior. The study of these interactions is of the utmost relevance for the design of future research aiming to understand how individuals think and act under physiological and pathophysiological conditions.
